# Complete genome sequence of *Stenotrophomonas acidaminiphila* strain ZCMU-Sa01, a cholesterol-metabolizing bacterium isolated from the feces of a healthy child

**DOI:** 10.1128/mra.00110-25

**Published:** 2025-06-23

**Authors:** Yu Lu, Sihan Feng, Qinchao Ding, Kexin Yang, Yiwen Zhang, Bin Ding

**Affiliations:** 1 School of Life Sciences, Zhejiang Chinese Medical University 70571, Hangzhou, China; 2 The School of Public Health, Zhejiang Chinese Medical University 668733, Hangzhou, China; 3 First Clinical Medical College, Zhejiang Chinese Medical University 623998, Hangzhou, China; Loyola University Chicago, Chicago, Illinois, USA

**Keywords:** genome sequence, *Stenotrophomonas acidaminiphila*, cholesterol-metabolizing, ZCMU-Sa01

## Abstract

*Stenotrophomonas acidaminiphila* strain ZCMU-Sa01, isolated from the feces of a 6-year-old healthy child, is a newly found bacterium that can grow with cholesterol and carbon dioxide as the carbon source in mineral salt medium.

## ANNOUNCEMENT

The 6-year-old healthy child’s fecal sample was collected in a collection pot at Zhejiang Chinese Medical University (30.1754°N, 120.1488°E) and diluted with a concentration gradient after adding glycerol to the filter and removing large particulate matter. After performing a concentration gradient dilution, the 10^−2^ to 10^−6^ dilutions were anaerobically cultured in expanded carbon dioxide deoxidized mineral salt media with cholesterol as the carbon source in a 37°C incubator for approximately 2–3 days. The enriched culture was again serially diluted and re-inoculated several times to obtain pure culture of the cholesterol-metabolizing strain. It is certain that the obtained strain, gram-negative (1), belongs to the genus *Stenotrophomonas acidaminiphila* after 16S rRNA gene Sanger sequencing (primers listed in Table 1). Thus, we named the strain ZCMU-Sa01.

**TABLE 1:** Primers used for 16S rRNA gene sequence (2)

Primer name	Sequence (5'→3′)	Target region
27F	AGAGTTTGATCMTGGCTCAG	V1–V9
1492R	GGTTACCTTGTTACGACTT	V1–V9

The purified strain ZCMU-Sa01 was anaerobically cultured in Luria-Bertani liquid medium at pH = 6, with additional 1% sodium chloride, and grown in a constant temperature shaker with 120 rpm at 42℃ for 48 h. Genome sequencing was performed on a PacBio Sequel and an Illumina NovaSeq 6000 (paired end, 2 × 150 bp). In detail, the genomic DNA was extracted with the sodium dodecyl sulfate/proteinase method (3). The genomic DNA was sheared into 10 kb fragments by centrifugation in g-TUBES (Covaris, Woburn, MA, USA), and these fragments were ligated with SMRT Bell sequencing adapters with SMRTbell Template Prep Kit (v.1.0) (PacBio, Menlo Park, CA, USA) (4) to build a 10 kb SMRT Bell Library. After being quantified with Qubit (v.2.0), the prepared library was then applied for PacBio sequencing. For Illumina sequencing, the DNA was sheared into 350 bp fragments, purified, end-repaired, and ligated with A-tailed adapters using the NEB NextUltra DNA Library Prep Kit for Illumina (NEB, USA) (5). The library insert sizes were measured using Agilent 2100 (6) to ensure quality. PacBio generated 360,803 raw reads (*N*_50_ = 10,376 bp), while Illumina produced 24,026,466 raw reads, achieving a coverage depth of 936×, and the genome completeness is 99.76%, assessed by CheckM (v.1.2.2) (7). Fastp (https://github.com/OpenGene/fastp) (8, 9) was used to remove PacBio and Illumina reads with lengths less than 500 bp, reads containing more than 10% N bases, and reads with adapters. Then PacBio reads were assembled by SMRT Link (v.5.0.1)(10, 11) into one contig with a contig *N*_50_ value of 3,875,194 bp. Subsequently, to correct the assembly error of PacBio assembly results, Pilon (v.1.22)(12) was used to remove reads with base mass values less than 20, depths less than 4, and depths greater than 1,000, based on the Illumina reads. If there was a 5,000 bp overlap of the two ends, then one end of the overlap was cut off to circularize the sequence. Coding sequence prediction and gene annotation were performed using the National Center for Biotechnology Information Prokaryotic Genome Annotation Pipeline (v.6.9) (13). Default parameters were used except where otherwise noted.

The complete genome comprised only a circular chromosome (3,848,402 bp, Guanine-Cytosine (GC) = 68.93%). Key features include 3,392 protein-coding genes (11 of which are related to cholesterol transport), 62 tRNAs, 9 rRNAs, 4 ncRNAs, and 3 prophage regions. No plasmids were detected.

## Data Availability

The genome sequence of *Stenotrophomonas acidaminiphila* strain ZCMU-Sa01 has been deposited in the GenBank under accession number CP178915, and raw reads are available with Sequence Read Archive accession numbers SRR32162630 and SRR32194861.The 16S rRNA gene sequence was deposited in the GenBank database with accession number PV342514.
